# The conjunction fallacy in rats

**DOI:** 10.3758/s13423-023-02251-z

**Published:** 2023-02-16

**Authors:** Valeria V. González, Sowgol Sadeghi, Linh Tran, Aaron P. Blaisdell

**Affiliations:** Department of Psychology, University of California Los Angeles, 1285 Franz Hall, Los Angeles, CA 90095-1563 USA

**Keywords:** Conjunction fallacy, Reasoning, Rats, Heuristics

## Abstract

Humans and other animals are capable of reasoning. However, there are overwhelming examples of errors or anomalies in reasoning. In two experiments, we studied if rats, like humans, estimate the conjunction of two events as more likely than each event independently, a phenomenon that has been called conjunction fallacy. In both experiments, rats learned through food reinforcement to press a lever under some cue conditions but not others. Sound B was rewarded whereas Sound A was not. However, when B was presented with the visual cue Y was not rewarded, whereas AX was rewarded (i.e., A-, AX+, B+, BY-). Both visual cues were presented in the same bulb. After training, rats received test sessions in which A and B were presented with the bulb explicitly off or occluded by a metal piece. Thus, on the occluded condition, it was ambiguous whether the trials were of the elements alone (A or B) or of the compounds (AX or BY). Rats responded on the occluded condition as if the compound cues were most likely present. The second experiment investigated if this error in probability estimation in Experiment 1, could be due to a conjunction fallacy, and if this could be attenuated by increasing the ratio of element/compound trials from the original 50-50 to 70-30 and 90-10. Only the 90-10 condition (where 90% of the training trials were of just A or just B) did not show a conjunction fallacy, though it emerged in all groups with additional training. These findings open new avenues for exploring the mechanisms behind the conjunction fallacy effect.

The conjunction fallacy has currently only been demonstrated in humans. The conjunction fallacy refers to an error in decision-making where the conjunction of two events is estimated to be more likely than each event by itself (Tversky & Kahneman, 1983). The conjunction fallacy is commonly referred to as the “Linda Problem” after the canonical example presented by Tversky and Kahneman (1983). This fallacy, the authors claimed, occurs because humans apply a representativeness heuristic to find the statement that supports the most stereotypical image of the example. By misapplication of the representativeness heuristic and availability heuristic, participants wrongly estimate that a conjunction event is more likely, leading to the conjunction fallacy (see Hertwig & Chase, 1998, for a review of findings).

Since then, many authors have proposed different accounts of the conjunction fallacy. What is common across all these accounts is that the phrasing of the problem induces an error in the reasoning process (Crupi et al., 2008; Sides et al., 2002). For instance, Sides et al. (2002) proposed that the conjunction fallacy might be due to people’s understanding of the word *probability*. They claimed that the common understanding of the word *probability* refers to evidential support—that is, instead of “what happens frequently,” which is the mathematical probability participants are tested on, people understand probability as “what is plausible” or “whether there is evidence” to support the statement (Crupi et al., 2008). In the original example of Linda, the initial statement gives support to the belief that the conjunction is true. To avoid this, Sides et al. (2002) used a betting paradigm in which participants needed to select the statement that they wanted to bet on to win a monetary prize, avoiding the use of words such as *probability*, *chance*, and *likelihood*. Despite the presentation of these new problems, the conjunction fallacy persisted.

More recently, the conjunction fallacy has been demonstrated in nonsocial situations that do not involve phrasing issues. Ludwin-Peery et al. (2020) had participants watch videos of a pink “ball” dropping towards a hole at the same time that a gray “ball” traveled across the screen such that it could collide with the pink ball. Different versions of the problem varied the size of the balls and the hole, the speed and location of the objects, and so forth. After watching each scenario, the participant judged the probability that either a single event would occur or a conjunction of two events would occur. Participants estimated the conjunction of two events as more likely, once again replicating the conjunction fallacy effect. Importantly, the conjunction fallacy in a physical reasoning task cannot be explained by any of the prior accounts that have been applied to nonphysical reasoning situations, and thus does not support the role of a representativeness heuristic as the only, or even primary cause of the conjunction fallacy. In that sense, the concept of “representativeness” seems less appropriate here because there is no salient category or schema to “activate.” Furthermore, unlike in the Linda problem where participants are given extra information in the form of Linda’s backstory that could bias participants toward a conjunction fallacy, the paradigms used by Ludwin-Peery et al. (2020) contain no extraneous biasing “backstory” information. Instead, participants merely watch physical events, such as potential collisions between moving objects or force interactions in static multi-object structures. Participants watched a video or observed a static scene, and yet rated the conjunction of events as more likely than sole events in all cases, thereby establishing the generality of the conjunction fallacy. The demonstration of a conjunction fallacy in these very basic physical reasoning tasks suggests that the conjunction fallacy is the product of general mechanisms of reasoning rather than specialized modules, such as in the social domain. Because animals share many general reasoning mechanisms with humans, the conjunction fallacy might also be found in nonhuman animals.

To our knowledge, the present report is the first attempt to explore the conjunction fallacy in a nonhuman species. In two experiments, we evaluated whether rats judge the occurrence of two events as more likely than the occurrence of each event independently. Rats first learned the relationship that auditory and visual cues had with the delivery of a food reward. Following acquisition, rats were presented with just the auditory cues. Their behavior reflected the expectation of food, and in turn, the expectation of food was determined by the belief that the cues currently presented on a test trial signaled the likelihood of food or no food. The critical manipulation involved occluding the visual cues on some test trials, thereby creating a perceptually ambiguous situation in which the rat could not determine if the auditory cue was the only cue present, or if one of the visual cues was also present though occluded. We have successfully used this procedure in prior studies to show that rats understand that an opaque metal shield can occlude perception of a visual cue when placed over the location of the cue’s source (i.e., the light bulb; see review by Blaisdell, 2019). Under certain conditions, rats behave as if they believe that the occluded visual cue is present. In the current study, behavioral responding at test can measure whether the rat believes that the occluded visual cue is certainly absent, possibly present, or certainly present.

## Experiment 1

### Method

#### Subjects

Sixteen female Long-Evans rats (*Rattus norvegicus*) acquired from Envigo (https://www.envigo.com/model/hsdblu-le) served as subjects. The rats had some experience with a Pavlovian procedure involving two lights that were qualitatively different from those used in this study and located in a different part of the conditioning chamber from the light used here. Rats were approximately 120 days old at the start of the experiment and were pair-housed in transparent plastic tubs with a wood shaving substrate in a vivarium maintained on a reverse 12-hr light cycle. Experiments were conducted during the dark portion of the cycle 7 days a week. A progressive food restriction schedule was imposed prior to the beginning of the experiment to maintain rats at 85% of their initial free-feeding weights. Water was always available in their home-cages. The procedures used in this, and the following experiment were conducted under approval and following the guidelines established by the IACUC of UCLA.

#### Apparatus

This experiment was conducted using eight experimental chambers, measuring 30 × 25 × 20 cm (L × W × H). Each chamber was housed in separate sound- and light- attenuating environmental isolation chests (ENV-008, Med Associates, Georgia, VT). The front and back walls and ceiling of the chambers were constructed of clear Plexiglas, the side walls were made of aluminum, and the floors were constructed of stainless-steel rods measuring 0.5 cm in diameter, spaced 1.5 cm center to center.

Each chamber was equipped with a pellet-dispenser (ENV-203-45, Med Associates) with a cup-type pellet receptacle (ENV-200R1M, Med Associates). When activated, one sucrose pellet was delivered into the cup. The opening of the cup was equipped with an infrared beam and a photodetector to record entries into the food niche. A retractable lever (ENV- 112CM) was located on the left side of the front panel, 2.1 cm above the stainless-steel grid floor.

A diffuse incandescent light (ENV-227M, Med Associates) was located on the center panel above the pellet receptacle, 6 cm above the grid floor. The light could be flashed at a rate of 2 Hz or presented as a steady light. These two types of light presentation served as Cue X and Cue Y, counterbalanced within groups. A speaker (ENV-224DM) on the ceiling of the chamber delivered a click train (4/s) 5 dB above background or a 3000-Hz tone 8 dB above background to serve as Cue A and Cue B, counterbalanced within-group. A 4-cm square solid stainless-steel cover, designed to mimic a covered light bulb, could be affixed to the metal wall of the chamber. A 62-dB background noise was produced from the combination of ventilation fans and a white noise generator in the room.

#### Procedure

Because animals had experience with the experimental chambers and the sucrose pellets, no magazine entry was needed prior to the training phase.

##### Lever press (Sessions 1–2)

On Session 1, rats received a 30-min session in which the left lever was available. Each lever press response was followed by delivery of a sucrose pellet into the food cup. On Session 2, an average of one of every four lever presses was reinforced (a variable ratio [VR-4] schedule).

##### Training (Sessions 3–15)

**Fig. 1:**
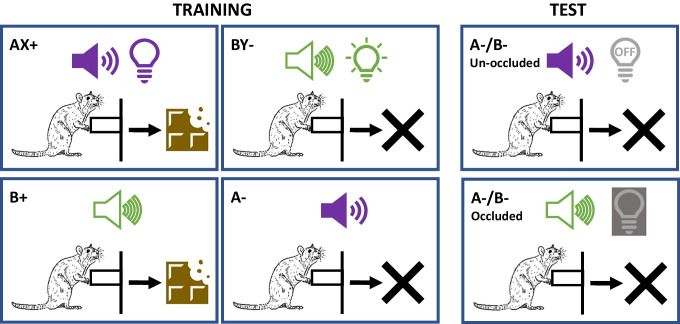
Design Experiment 1. *Note*. Training: A and B correspond to auditory cues (a tone and a white noise, counterbalanced presented from the same speaker). X and Y correspond to visual cues (a steady or flashing light presented from the same bulb). + and − indicate whether lever presses during the cue presentation were reinforced with food or not, respectively. Test: Auditory cues were presented with the light bulb explicitly off or occluded by a metal cover

On Sessions 3–15, all subjects received daily training sessions lasting approximately 120 mins. Each session consisted of 40 trials, in which all rats received 10 presentations of each element A- and B+, and each compound AX+ and BY-, in quasirandom order (see Fig. 1). A and B were auditory cues (a tone and a white noise generated from the same speaker), whereas X and Y were visual cues (steady or flashing light presented in the same light bulb); the specific visual or auditory cue assigned to each element or compound was counterbalanced across subjects, orthogonally with respect to each cue type. Lever presses made during the 30-s presentations of BY- or A- were not reinforced, whereas lever presses made during the 30-s AX+ compound or of B+ were reinforced with 1 sucrose pellet on a VR-4 schedule. Reinforcement could be delivered multiple times on a given trial. Lever pressing was recorded during a 30-s period before the start of each trial (preCue) and during each 30-s trial (Cue). A variable 2-minute ITI separated the trials. Mean elevation scores were calculated for each session (lever presses during 30-s Cue duration minus 30-s preCue lever presses) for the four trial types.

##### Test A, B (Sessions 16–17)

On Sessions 16 and 17, rats received eight separate presentations of Cues A and B. On the first test session, for half of the animals, the bulb was uncovered and remained off during the entire test session though the metal occluder was placed on the panel wall next to the bulb, while for the remaining rats the bulb was occluded with the metal cover during the session (and the light was off). Each rat received a similar test session the following day, but with the opposite state of the bulb (occluded/unoccluded) from the first test session. Notice that for the unoccluded condition, the cover was presented at the same height but next to the light, thereby producing a similar added feature to each test condition. A variable 2-minute ITI separated the trials. Lever presses were recorded in the same fashion, though no reinforcement was delivered throughout the session (i.e., nonreinforced probe tests). Mean elevation scores (lever presses during 30-s Cue duration minus 30-s preCue lever presses) were calculated for A and B for each test condition (occluded/unoccluded).

##### Statistical analysis

All statistical analyses were conducted using repeated-measures (RM) analyses of variance (ANOVA) of elevation scores as the dependent variable, and Session, Cue type, Test Condition (Occluded or Unoccluded) as repeated measures. Post hoc analyses were performed using Holm correction. The software JASP was used for all analysis (JASP Team, 2022)

### Results

**Fig. 2:**
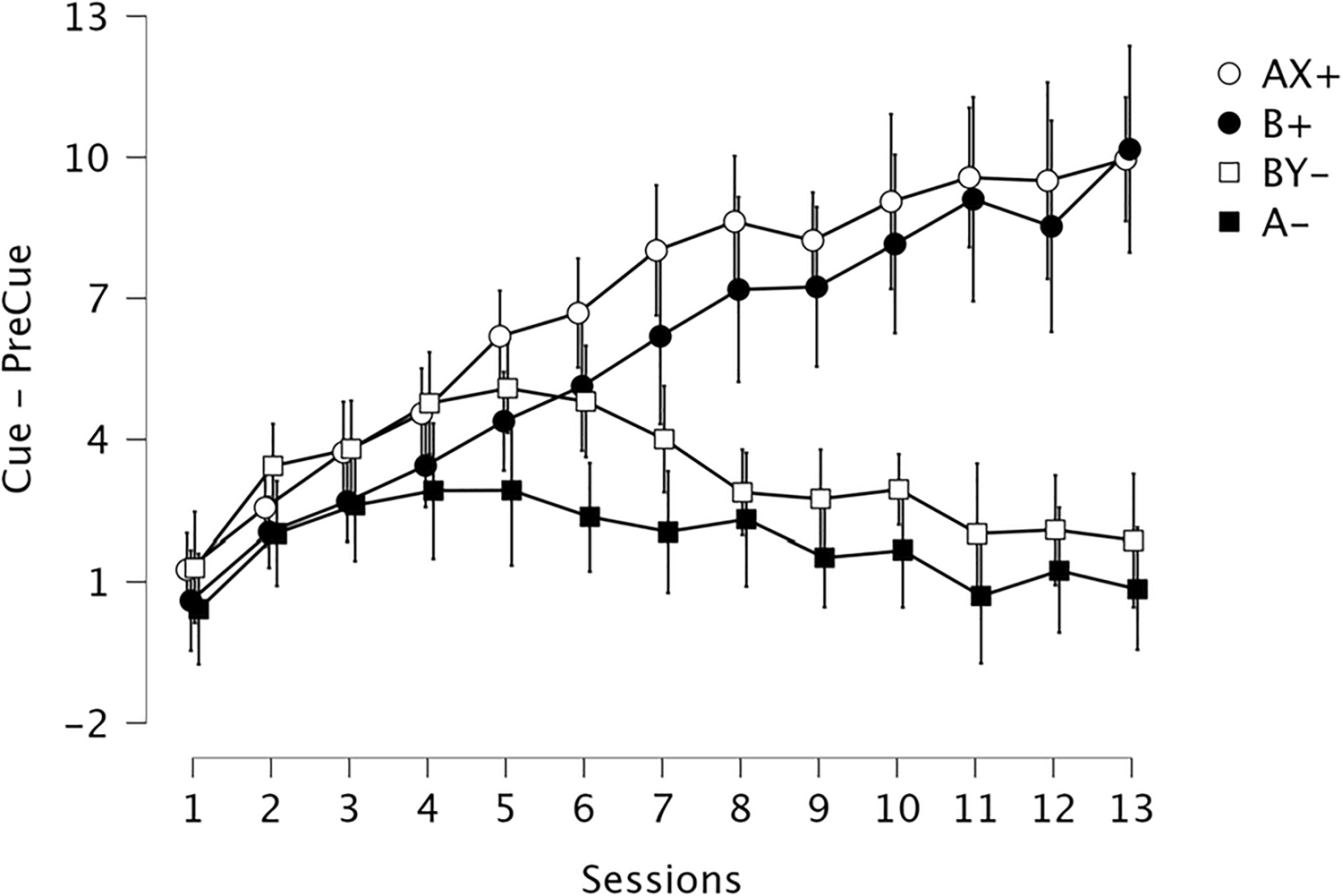
Acquisition performance by cue for Experiment 1. *Note.* Elevation scores (Cue − PreCue) were calculated using the total number of lever presses during the 30-s Cue duration minus the total level presses during the 30-s before the presentation of each cue for each session of training. Error bars depict 95% Confidence Intervals

By the end of acquisition, responding was much higher on reinforced trials compared with nonreinforced trials showing that rats had mastered the discrimination (Fig. 2). The ANOVA revealed an effect of Session, *F*(12, 180) = 19.38, *p* < .001, ɳ^2^ = 0.128, and of Cue, *F*(3, 45) = 25.48, *p* < .001, ɳ^2^ = 0.273, and a Session × Cue interaction, *F*(36, 540) = 18.39, *p* < .001, ɳ^2^ = 0.186. Responding did not differ between reinforced stimuli (AX+ and B+, *p*_*Holm*_ = .123, *d* = 0.393) or between nonreinforced stimuli (BY-, A-, *p*_*Holm*_ = .067, *d* = 0.548), while elevation scores on AX+ and B+ trials were higher than on BY- and A- trials (*p*s < .001, *d* > 0.999).

**Fig. 3:**
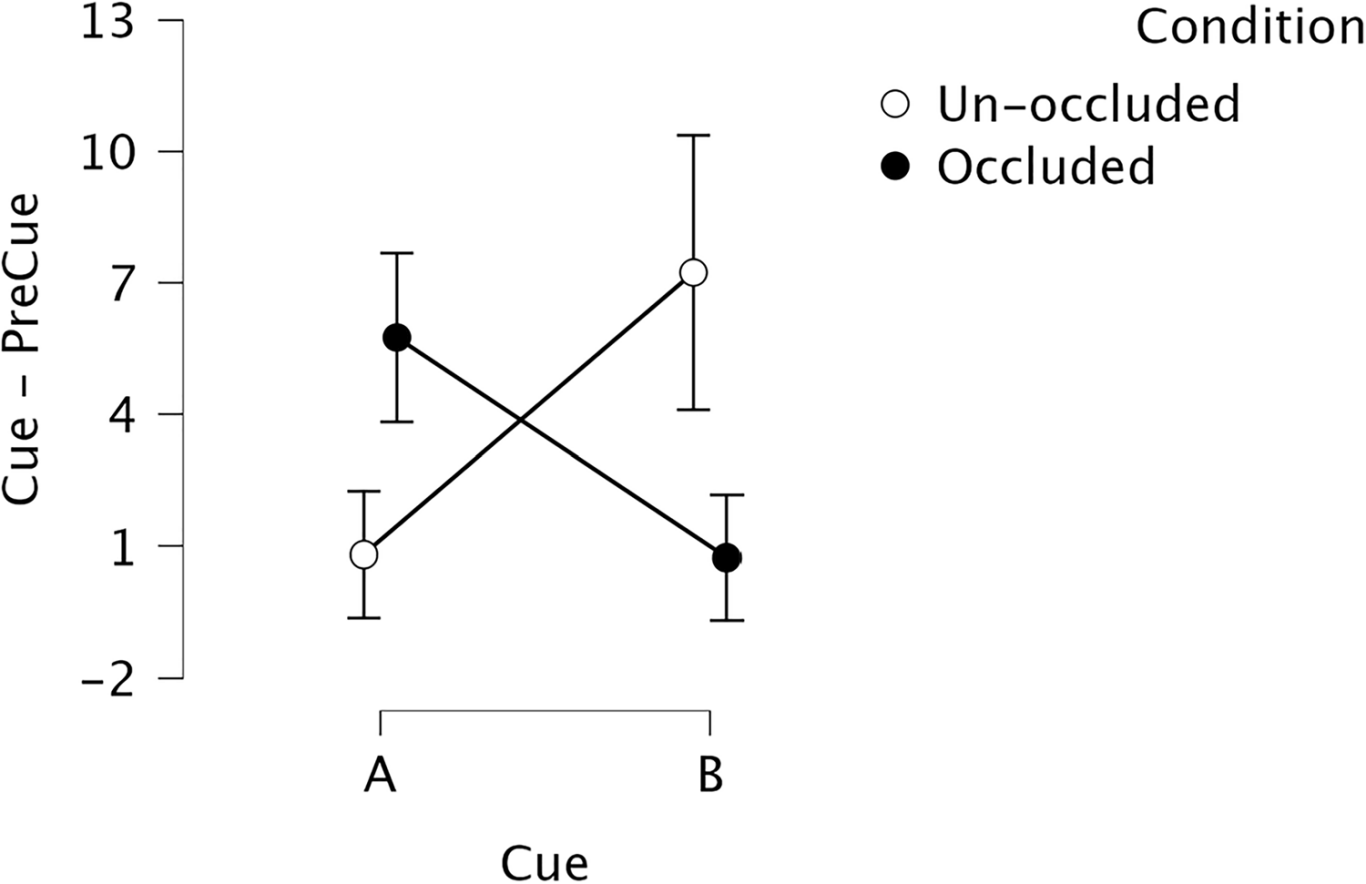
Test Experiment 1. *Note.* Elevation scores (Cue − PreCue) were calculated using the 30-s cue duration minus the 30-s before the onset of the cue. Auditory cues A and B were presented without reinforcement. The filled circle refers to trials in which the light bulb, where cues X and Y were presented during training, was explicitly off. The empty circle corresponds to trials with the light bulb occluded. Error bars depict 95% Confidence Intervals

Figure 3 shows the results of the test sessions for Cues A and B with the light Occluded or Unoccluded. During Unoccluded tests, rats made few lever press responses when A was presented with the light off, similar to responses during A- trials during training; and many responses when B was presented with the light off as on B+ trials during training. Interestingly, lever pressing to A and B with the light occluded showed the opposite pattern, suggesting that the rats “believed” that Light X was on behind the occluder whenever Cue A was presented, and that Light Y was on behind the occluder whenever Cue B was presented. ANOVA revealed no effect of Condition, *F*(1, 15) = 0.76 *p* = .398, ɳ^2^ = 0.008; nor Cue, *F*(1, 15) = 0.60, *p* = .451, ɳ^2^ = 0.006; but did find a Cue × Condition interaction, *F*(1, 15) = 25.54, *p* < .001, ɳ^2^ = 0.422. When A or B were presented with the light explicitly off (Unoccluded condition), responses were higher for B than A (*p*_*Holm*_ < .001), as during training. Elevation scores during Cue A with the light occluded were higher than with the light unoccluded (*p*_*Holm*_ = .007). Similarly, elevation scores during Cue B with the light occluded were lower than with the light unoccluded (*p*_*Holm*_ < .001). Responses during Cue A with the light occluded were higher than during Cue B (*p*_*Holm*_ = 0.007), and responses during Cue A with the light unoccluded were lower than during Cue B (*p*_*Holm*_ < .001). Finally, responses to Cue A with the light occluded were not different from responses to Cue B unoccluded (*p*_*Holm*_ = .508) neither when Cue B had the occluded light compared with responses with Cue A unoccluded (*p*_*Holm*_ = .956); this last result indicates that, as in training, AX and B had a similar association with reinforcement, as BY and A were similarly associated with no reinforcement. To assess evidence in support of the null hypothesis of no difference between A occluded and B unoccluded and between A unoccluded and B occluded, we conducted a Bayes Factor for each comparison. There was moderate evidence that responding during A occluded was not different than during B unoccluded, BF_10_ = .264 (error = .012), and moderate evidence that responding during A unoccluded was not different than during B occluded, BF_10_ = .345 (error = .015).

The results suggest that rats assumed the presence of the light on during occluded trials was more likely than the light off. This could be considered as a first demonstration of a conjunction fallacy in a nonhuman animal. We have previously shown that rats can form mental images about absent events (e.g., Blaisdell et al., 2009; Fast, Biedermann, & Blaisdell, 2016a; Fast & Blaisdell, 2011; Fast, Flesher, et al., 2016b; Waldmann et al., 2012). For example, after tone–light pairings, presenting the tone can retrieve the memory of the light. If the light is occluded by a metal cover, the rat can act as if the light is actually present, consistent with their prior training. What is novel and surprising in the current study is that each auditory cue was paired with the light on half the trials and was presented alone on the remaining trials. Yet rats acted as if they were confident that on the occluded trials, the occluded light was present. This can be explained in two different ways. We can understand the result as an error in reasoning: A rational rat should presumably believe that there is a 50% chance on any given test trial with an auditory cue that the associated visual cue was present. Instead, rats acted nonrationally, as if on occluded trials with A, X was more likely to be present than absent, and on occluded trials with B, Y was more likely to be present than absent. Alternatively, rats might believe that during occluded trials the light was always on because they already experienced all unoccluded trials with the light off; thus, the rat can assume that all remaining trials the light should be on to match training experience. However, we believe this explanation is unlikely because rats had separate sessions with occluded and unoccluded trials, and even if true, this explanation could only apply to half of the rats that received the unoccluded test trials first. For the rats that received the occluded test trials first, there would be no basis for this kind of “counting trial” explanation. Furthermore, it is remarkable that the bias towards believing the compound cues were present on occluded trials was equally strong whether the compound had been rewarded or unrewarded during training, suggesting that the effect is not due to any simple effects of reinforcement.

## Experiment 2

Experiment 2 was designed to reduce or eliminate the conjunction fallacy. Three groups of rats received the same training as in Experiment 1 but differed in the ratio of element-compound trials: Group 50-50 (same as Experiment 1), Group 70-30, and Group 90-10. If rats encode the frequency of events and use that information to judge the probability of an element or compound on an occluded test trial, then increasing the ratio of element trials should reduce or eliminate the conjunction bias at test.

### Method

#### Subjects

Thirty female Long-Evans rats (*Rattus norvegicus*) acquired from Envigo served as subjects. Subjects were approximately 90 days old at the start of the experiment. Subjects were maintained in the same conditions as Experiment 1. The experiment was conducted during the dark portion of the cycle 5 days a week.

#### Apparatus

This experiment was conducted in two rooms, using 16 experimental chambers, the same as described in Experiment 1.

An LED light was located on the left panel above the lever 6 cm above the grid floor. The light could be flashed at a rate of 2 Hz or presented as a steady light. The steady and flashing light modality served as the two visual cues X and Y, counterbalanced within groups. A speaker (ENV-224DM) on the ceiling of the chamber delivered a 3000-Hz tone and a white noise, 8 dB above background, to serve as A and B auditory cues for training, counterbalanced within-group. A 4-cm square solid stainless-steel cover, designed to mimic a covered light bulb, could be affixed to the metal wall of the chamber. A 62-dB background noise was produced from the combination of ventilation fans and a white noise generator in the room.

#### Procedure

##### Magazine training (Session 1)

On Session 1, rats were trained to eat pellets from the food cup by delivering one pellet every 20 ± 15 s (actual intertrial interval [ITI] values = 5, 10, 15, 20, 25, 30, and 35 s), until they consumed 30 pellets.

##### Lever press (Sessions 2–4)

On Session 2 and 3, rats received a 30 min session in which the left lever was available. Each lever press response was followed by delivery of a sucrose pellet. Rats that did not press more than 30 times by the second session of lever press training received some extra trials of hand-shaping (i.e., the experimenter manually administrate pellets to shape the rat’s behavior) until they were reliably lever pressing. On Session 4, an average of one of every four lever presses was reinforced (VR-4 schedule).

##### Training 1 (Sessions 5–17)

On Sessions 5–17, all subjects received daily conditioning sessions lasting approximately 120 mins. As in Experiment 1, each session consisted of 40 trials, in which rats received presentations of AX+, A-, BY- and B+ in quasirandom order (see Fig. 1). The frequency of each trial type depended on the group (See Table 1). Group 50-50 (*n* = 10) was a replication of Experiment 1; therefore, the number of each trial type was equal within each session. For Group 70-30 (*n* = 10), rats received 14 presentations of each element B+ and A-, and 6 presentations of each compound AX+ and BY- per session. For Group 90-10 (*n* = 10), rats received 18 presentations of each element B+ and A-, and two presentations of each compound AX+ and BY- per session. As in Experiment 1, lever presses made during presentations of BY- or A- were not reinforced, whereas lever presses made during presentations of AX+ and B+ were reinforced with 1 sucrose pellet on a VR-4 schedule. Reinforcement could be delivered multiple times on a given trial. Lever pressing was recorded as in Experiment 1.

**Table 1:** Design Experiment 2

Group	Training (1 & 2)	Test (1 & 2)	
50-50	10AX+/10A-/10BY-/10B+	8A-/8B-	X/Y occluded or unoccluded
70-30	6AX+/14A-/6BY-/14B+		
90-10	2AX+/18A-/2BY-/18B+		

Training: A and B correspond to auditory cues (a tone and a white noise, counterbalanced presented in the same speaker). X and Y correspond to visual cues (a steady or flashing light presented in the same bulb). Test: Auditory cues were presented with the light bulb explicitly off or occluded with a metal cover. + and − indicate if lever presses during the cue presentations were going to be reinforced or not, respectively. The number indicates the total of presentations of each cue in a session

##### Test 1 (Sessions 18–19)

On Sessions 18 and 19, rats received eight separate presentations per session of A and B as in Experiment 1, with test order counterbalanced within group.

##### Training 2 (Sessions 20–22)

On Sessions 20–22, subjects received extra sessions of training as described above.

##### Test 2 (Sessions 23–24)

On Sessions 23 and 24, rats received another two sessions as described for Test 1.

##### Statistical analysis

All statistical analyses were conducted using mixed ANOVAs of elevation scores as the dependent variable, Group (50-50, 70-30, and 90-10) as a between-group factor, and Session, Cue type, Test Condition (Occluded or Unoccluded) as repeated measures. Post hoc analyses were performed using Holm correction. The software JASP was used for all analysis (JASP Team, 2022).

### Results

**Fig. 4:**
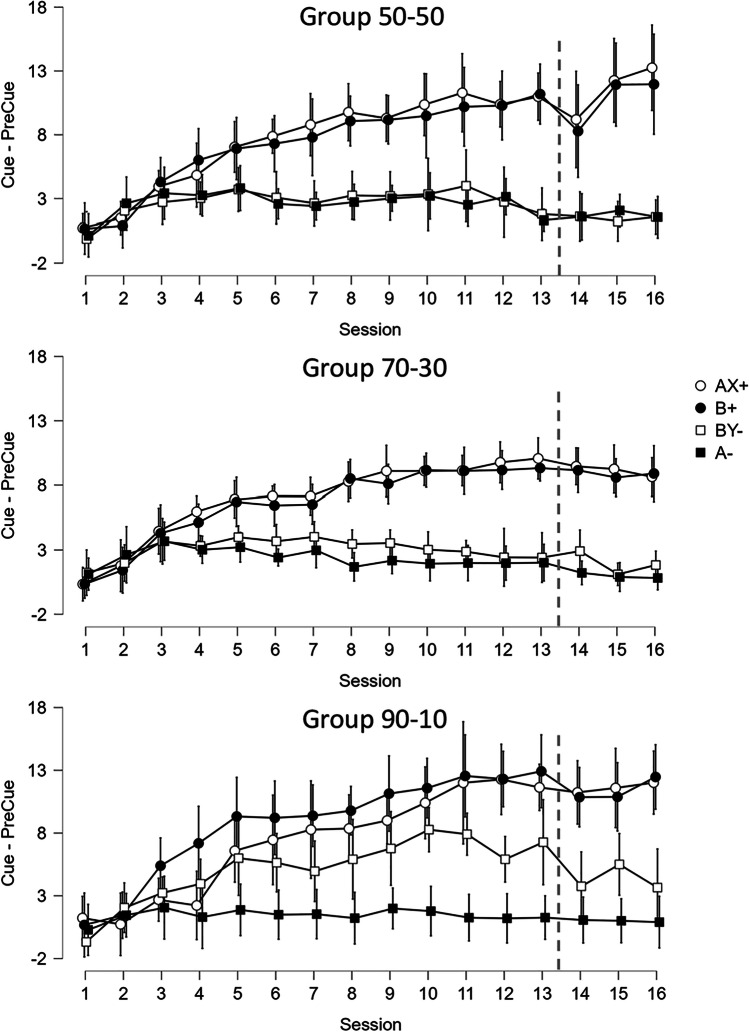
Acquisition of Training 1 and 2 by group of Experiment 2. *Note.* Elevation scores were calculated by lever press responses prior to cue onset subtracted from lever press responses during the cue. The letters indicate the cues trained, in which A and B correspond to auditory cues and X and Y to visual cues. + and − in the legend indicate the cues associated with reinforcement and nonreinforcement, respectively. Each panel corresponds to one experimental group. The dashed line indicates when Test 1 was performed, thus, the next three sessions (14–16) correspond to Training 2. Error bars correspond to 95% Confidence Intervals

The top and middle panels of Fig. 4 show a replication of the pattern of acquisition from Experiment 1 for Groups 50-50 and 70-30 by Session 13 (same number of acquisition sessions as in Experiment 1). By contrast, in Group 90-10 (bottom panel) elevation scores increased for BY- trials and never came down by Session 13 despite not being reinforced. ANOVA found main effects of Session, *F*(12, 348) = 64.30, *p* < .001, ɳ^2^ = 0.159; and Cue, *F*(3, 87) = 44.89, *p* < .001, ɳ^2^ = 0.207, but not of Group, *F*(2, 29) = 0.47, *p* = .630, ɳ^2^ = 0.006. We also found Session × Group interactions, *F*(24, 348) = 2.09, *p* = .002, ɳ^2^ = 0.010, and between Session and Cue, *F*(36, 1044) = 24.40, *p* < .001, ɳ^2^ = 0.088, but no other interactions were found.

**Fig. 5:**
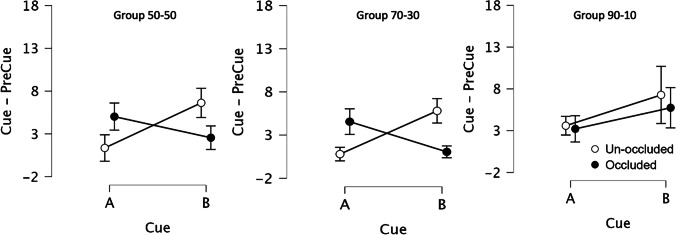
Results Test 1 by Group. *Note.* Elevation scores (Cue − PreCue) were calculated for the auditory cues A and B with the light bulb explicitly off (condition unoccluded, empty circles in the figure) and with the bulb covered with a metal piece (condition occluded, filled circles). From left to right, the panels show responses for Group 50-50 (left), Group 70-30 (middle) and Group 90-10 (right). Error bars correspond to 95% Confidence Intervals

Figure 5 shows the results of Test 1 for Cues A and B in conditions Occluded and Unoccluded by group. Group 50-50 (left panel) and Group 70-30 (middle panel) replicated the pattern of responding observed in Experiment 1. By contrast, the pattern of responding was the same for occluded and unoccluded tests in Group 90-10 (right panel), which was how rats responded to these cues during training. ANOVA found main effects of Cue, *F*(1, 29) = 15.91, *p* < .001, ɳ^2^ = 0.072, and Group, *F*(2, 29) = 6.01, *p* = .007, ɳ^2^ = 0.062, but no effect of Condition, *F*(1, 29) = 1.02, *p* = .322, ɳ^2^ = 0.007. The only interactions found were between cue and condition, *F*(1, 29) =70.90, *p* < .001, ɳ^2^ = 0.198, and between Cue × Condition × Group, *F*(2, 29) = 11.98, *p* < .001, ɳ^2^ = 0.067, suggesting that the pattern of test results for Group 90-10 contrasted from those found in Groups 50-50 and 70-30. Therefore, we conducted a second set of ANOVAs, one pooling the data from Groups 50-50 and 70-30 and the other isolating outlier group 90-10. ANOVA on Groups 70-30 and 50-50 found a significant Cue × Condition interaction, *F*(1, 20) = 225.47, *p* < .001, ɳ^2^ = 0.599. As in Experiment 1, responses to A were lower than responses to B when the light was explicitly off (*p*_*Holm*_ < .001), responses to A when the light was occluded were higher than when the light was off and unoccluded (*p*_*Holm*_ < .001), the contrary was true with B occluded versus unoccluded (*p*_*Holm*_ < .001), and A and B occluded were different (*p*_*Holm*_ < .001). Finally, A unoccluded compared with B occluded or B unoccluded compared with A occluded were not different (*p*_*Holm*_ = .316, *p*_*Holm*_ = .076). The bayes factor was calculated for the last two comparisons, finding a BF_10_ = .487 (error = .023) and BF_10_ = .909 (error = .024), respectively. The ANOVA on Group 90-10 found only a main effect of Cue, *F*(1, 10) = 15.66, *p* = .003, ɳ^2^ = 0.224, showing that the group learned the trained discrimination but the use of a cover over the light did not influence their behavior.

Only in the 90-10 training condition were we able to attenuate the conjunction bias found in the 50-50 condition of Experiments 1 and 2, and the 70-30 condition of Experiment 2. It is striking how severely we had to skew the ratio of training in favor of element versus compound trials to attenuate this bias. Nevertheless, the manipulation of the ratio of element to compound trials is also confounded with the absolute number of compound trials received during training, with rats in Group 90-10 receiving far fewer compound trials than rats in the other two conditions. Perhaps there were an insufficient number of AX+ and/or BY- trials in Group 90-10 to induce the conjunction fallacy. To assess this, we implemented another three sessions of training followed by testing. If the conjunction bias requires a threshold number of compound trials to emerge, then additional training might eventually lead the rats in Group 90-10 to show the conjunction bias. As seen in Fig. 4 after three additional sessions of acquisition, lever pressing on BY- trials in Group 90-10 began to look more like they did in the other two groups and are more similar to the response levels observed on A- trials.

**Fig. 6:**
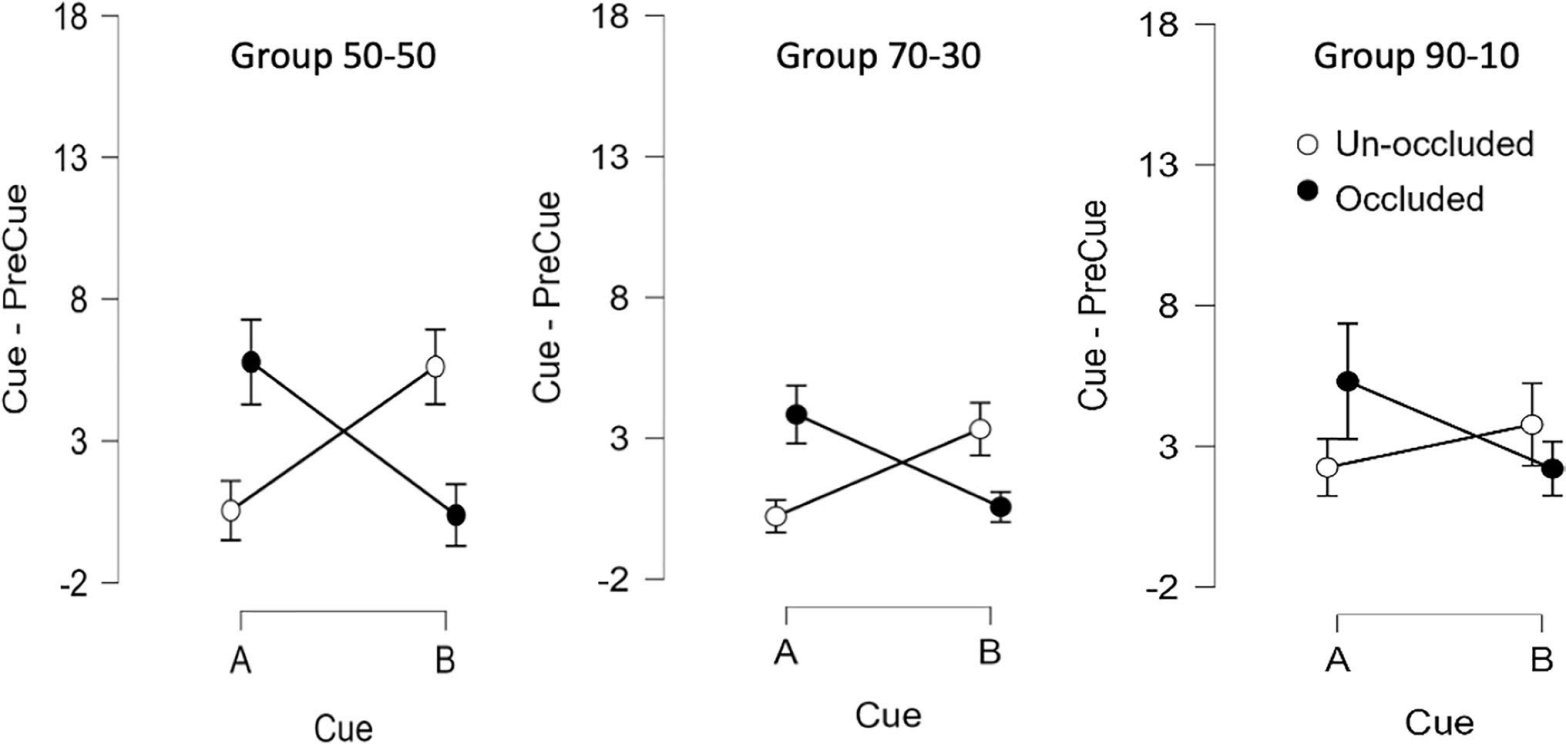
Results Test 2 by group. *Note.* Elevation scores (i.e., lever presses during 30-s Cue duration minus lever presses during 30-s PreCue duration) were calculated for the auditory cues A and B with the light bulb explicitly off (condition unoccluded, empty circles in the figure) and with the bulb covered with a metal piece (condition occluded, filled circles). From left to right, the panels show responses for Group 50-50 (left), Group 70-30 (middle), and Group 90-10 (right). Error bars correspond to 95% Confidence Intervals

After additional training, the pattern of test results in Group 90-10 were similar to the other two groups (Fig. 6); with rats behaving during the occluded condition as if the light that had been paired with the test cue was on. ANOVA replicated the pattern of results from Test 1 except that no effect of Cue, *F*(1, 29) = 1.32, *p* = .261, ɳ^2^ = 0.004; nor of Group, *F*(2, 29) = 2.33, *p* = .115, ɳ^2^ = 0.046 were found. The same interaction as Test 1 was significant (*p* < .007). Post hoc tests replicated the Experiment 1 pattern, including in Group 90-10 (*p*_*Holm*_ < .001). The Bayes Factor analysis revealed a BF_10_ = .299 (error = .006) and BF_10_ = .559 (error = .015), respectively for comparisons between A occluded and B unoccluded and A unoccluded and B occluded for Group 90-10, providing evidence of no difference in responding between these conditions.

## Discussion

This is the first report of a conjunction fallacy effect in a nonhuman animal, specifically the laboratory rat. In Experiment 1, rats learned that lever pressing during one auditory cue was reinforced when presented alone (B+) but was not reinforced when presented in compound with a light (BY-), while lever pressing during another auditory cue was not reinforced when presented alone (A-) but was reinforced when presented in compound with a different light (AX+). During the occluded tests when the light bulb was occluded by a metal shield, rats behaved as if the compound auditory–visual cue was present. Because the correlation between light and auditory cue during training was 50%, rats behaved as if they were certain that the occluded light was on when they heard the auditory cue at test. This is a violation of the prior probabilities and demonstrates a conjunction fallacy.

In Experiment 2, we attempted to attenuate or eliminate the conjunction fallacy by increasing the ratio of element to compound trials during acquisition. Compared with replicating the conjunction fallacy in Group 50-50 in Experiment 2, increasing the ratio to 70-30 in favor of element trials did not reduce this bias. Only in Group 90-10, where 90% of trials during acquisition were of each auditory element alone, did the conjunction fallacy effect attenuate. Nevertheless, with an additional three sessions of acquisition following the first test, the conjunction fallacy emerged even in Group 90-10. This suggests that the total number of compound trials during acquisition may play an important role in expressing the conjunction fallacy. However, it was not sufficient to prevent the conjunction fallacy from emerging.

The finding of a conjunction fallacy in nonhuman animals supports the conclusion of recent research reporting the conjunction fallacy in humans in nonsocial settings (Ludwin-Peery et al., 2020). The strong conjunction fallacy demonstrated in both experiments is at odds with some of the prior demonstrations from our laboratory on decision-making under conditions of perceptual ambiguity. For example, Fast and Blaisdell (2011) trained rats to lever press in the presence of one of two lights presented alone (X+, Y+) but not when both lights were presented in compound (XY-). At test, occluding one of the lights while presenting the other light resulted in rats acting as if the occluded light *might* be present. While this result is consistent with the results reported here, the strength of the effect was weaker. Specifically, rats responded at an intermediate level between the level of responding supported by a strong predictor of reinforcement and the level of responding supported by a strong predictor of the absence of reinforcement. Some of our other reports under conditions of perceptual ambiguity have found results more in line with our current results, that is, where responding controlled by the occluded event is equivalent to levels of responding if it were perceptually present (e.g., Fast, Biedermann, & Blaisdell, 2016a; Fast, Flesher, et al., 2016b).

It has been suggested that to interact successfully with our environment, we need to be able to reason flexibly about how situations might developed (similar to physical simulations in video games; Battaglia et al., 2013). In the reported example about physical reasoning (Ludwin-Peery et al., 2020), the authors discussed that the bias might be explained by a bias in *mental simulation*. Indeed, the results reported here with rats seem to give support to the idea of a bias in mental simulation. In a recent study by Bass et al. (2021), they proposed that partial mental simulation explains the conjunction fallacy without involving language. The authors argue that full simulations are costly for the brain, therefore organisms operate by creating partial simulations involving relevant aspects of the current situation (cf. the argument that humans and rats use minimal rational models to make decisions about causal relations; Waldmann et al., 2008). This account can explain the fallacy in intuitive physical reasoning as well as the results of our experiments. In nature, organisms typically do not have access to all the relevant information about a situation in which a decision is being made. It is possible there are heuristic biases to believe something is present (i.e., fill in missing information) when only partial information is available (e.g., a lion only sees the head of the antelope above the tall grass; a squirrel only sees part of a nut covered by leaves). If humans and other animals create partial simulations to facilitate decision-making, we might expect systematic biases, such as the conjunction fallacy.

The strength of the conjunction fallacy has implications for psychopathological conditions that involve symptoms of delusions and hallucinations. A false belief (delusion) or perceiving events in their absence (hallucinations) are characteristic symptoms of schizophrenia and certain forms of anxiety, such as obsessive-compulsive disorder (OCD), as well as posttraumatic stress disorder (PTSD). The role of associative processes in eliciting mental imagery in humans is now well established and appears to play a role in hallucinations (Powers et al., 2017) and in clinical settings (Dadds et al., 1997). Rodents may serve as a useful animal model in which to study such phenomena. For example, Kim and Koh (2016) found that genetic knockout mice that serve as a typical model of schizophrenia showed impaired reality monitoring, that is, the ability to distinguish real from imagined events, thus serving as a behavioral assay for the positive symptoms of schizophrenia. An animal model for the conjunction fallacy could also be useful to interrogate the mechanisms by which fear learning can occur to associatively retrieved events, even when those events are not actually present, as has been shown in humans (Mueller et al., 2019).
